# Magnetic Resonance Imaging of the Newborn Brain: Automatic Segmentation of Brain Images into 50 Anatomical Regions

**DOI:** 10.1371/journal.pone.0059990

**Published:** 2013-04-02

**Authors:** Ioannis S. Gousias, Alexander Hammers, Serena J. Counsell, Latha Srinivasan, Mary A. Rutherford, Rolf A. Heckemann, Jo V. Hajnal, Daniel Rueckert, A. David Edwards

**Affiliations:** 1 Faculty of Medicine, Imperial College London, and Medical Research Council Clinical Sciences Centre, Hammersmith Hospital, London, United Kingdom; 2 Department of Computing, Imperial College London, South Kensington, London, United Kingdom; 3 The Neurodis Foundation (Fondation Neurodis), CERMEP – Imagerie du Vivant, Lyon, France; 4 Centre for the Developing Brain, King’s College London, London, United Kingdom; University of Barcelona, Spain

## Abstract

We studied methods for the automatic segmentation of neonatal and developing brain images into 50 anatomical regions, utilizing a new set of manually segmented magnetic resonance (MR) images from 5 term-born and 15 preterm infants imaged at term corrected age called ALBERTs. Two methods were compared: individual registrations with label propagation and fusion; and template based registration with propagation of a maximum probability neonatal ALBERT (MPNA). In both cases we evaluated the performance of different neonatal atlases and MPNA, and the approaches were compared with the manual segmentations by means of the Dice overlap coefficient. Dice values, averaged across regions, were 0.81±0.02 using label propagation and fusion for the preterm population, and 0.81±0.02 using the single registration of a MPNA for the term population. Segmentations of 36 further unsegmented target images of developing brains yielded visibly high-quality results. This registration approach allows the rapid construction of automatically labeled age-specific brain atlases for neonates and the developing brain.

## Introduction

Anatomical structures can be segmented in biological images by transfer of voxel labels from an analogous image previously segmented into anatomical regions, or atlas [1]. This requires an accurate alignment and correspondence of structurally equivalent regions between the atlas and the target image usually achieved using non-rigid registration [2], [3], [4], [5], [6]. Segmentation methods often first register the atlas to the target image and then segment the target image into anatomical structures based on transferred information [5], [7], [8], [9], [10], [11], [12], although registering multiple atlases to the same target with subsequent fusion of different segmentations will frequently improve the final segmentation result, compensating for nonsystematic errors in single registrations [13], [14], [15], [16], [17].

Magnetic Resonance (MR) images of the brains of newborn infants have been particularly difficult to segment [18] due to: the low tissue contrast; signal inhomogeneity; intersubject differences due to the rapid development of the brain, especially in white matter (WM) structures [19]. The development of automatic segmentation methods has been further hindered by the lack of gold standard data for comparison and validation [20].

In this paper we present two methods for the automatic segmentation of neonatal and developing brain MR images into 50 regions of interest (ROI) utilizing a new set of manually defined neonatal atlases called ALBERTs [21]. The first approach is based on fusion of anatomical prior information from various neonatal atlases. The second approach is based on propagation of labels from a maximum probability neonatal ALBERT (MPNA). For both methods we evaluated the performance of different atlases and MPNAs and compared the results to the gold-standard manual segmentations.

## Materials and Methods

### Image Acquisition

MR images were acquired using a 3.0 Tesla Philips Achieva scanner (Philips Medical Systems, Best, The Netherlands). The technical characteristics of the scans, as well as detailed demographics, can be found in our previous work [21]. T1-weighted magnetization prepared rapid-acquisition gradient echo volumes in the sagittal plane were acquired with an echo time of 4.6 ms and repetition time 17 ms; 124–150 sagittal slices of 1.6 mm thickness were acquired with a 210 mm field of view, a flip angle of 30°, and a 256×256 matrix, resulting in voxel sizes of 0.82×0.82×1.6 mm^3^. We used the following data sets:

- 15 preterm neonates scanned at term (eight female), with a median gestational age at birth of 29 weeks (range 26–35 weeks) and a median gestational age at the time of the scan of the subjects of 40 (37–43) weeks, manually segmented [21]. Two were twins with a gestational age of 29 weeks, scanned at 40 weeks.
- Five term control neonates (two female), with a median age at scan of 41 (39–45) weeks, manually segmented [21].
- 36 preterm neonates scanned at birth that had not been manually segmented (sixteen female), with a median gestational age at birth of 29 (24–36) weeks.

Approval for scanning the subjects had been obtained from the Hammersmith Hospital Research Ethics Committee, and written informed consent obtained prior to scanning. Post-processing of anonymised scan data that had been acquired for clinical purposes did not require individual consent from the individuals who had been scanned.

### MR Image Pre-processing

T1-weighted 3D image volumes were obtained in DICOM format and converted to the NIfTI format using the UCLA Laboratory of Neuro-Imaging’s Debabeler Software (www.loni.ucla.edu/Software/Debabeler). The image matrix was reduced in superior, anterior, posterior, and lateral directions to contain five empty slices (5×0.82 = 4.1 mm) after the last slice containing skin. Inferiorly, five empty slices (5×1.6 = 8 mm) were added after the last slice where the posterior floor of the skull was visible. The reduction in matrix size simplified the subsequent bias correction step in that inferior extracranial signal would not have to be considered. The padding around the skull was maintained because our previous work had shown the skull to be an essential landmark for successful registration in young children [16], [17]. T1-weighted image volumes were corrected for non-uniformity using the FAST Software from the FMRIB Software Library (FSL version 4, [22]). T1-weighted images were resampled, creating isotropic voxels of 0.82×0.82×0.82 mm^3^ using windowed sinc interpolation, to make them compatible with Analyze AVW 8.1 software. Re-orientation of the sagittal T1 volumes was performed with the horizontal line defined by the anterior and posterior commissures (AC-PC orientation) and the sagittal planes parallel to the midline [23]. We reduced the number of interpolation steps during reorientation by coregistration of the native images onto the re-orientated versions of themselves. This was performed using a method based on normalised mutual information and 7^th^ degree B-spline interpolation. Coregistration was performed using SPM2 (Statistical Parametric Mapping, Wellcome Trust Centre for Neuroimaging, UCL, London) [24], [25] under Matlab version 6.5 (Mathworks Inc, Sherborn, MA, USA). A 16-bit voxel depth was maintained throughout the process.

### Delineation, Manual Segmentation, and Nomenclature

The MR images had been manually segmented into 50 ROIs each, covering the whole brain, using newly created protocols established according to previously described principles [26], [27], [28]. Each voxel belongs to one ROI only, and their ensemble thus constitutes a brain atlas: a label-based encephalic ROI template (ALBERT), as described in detail in Gousias et al., 2012 [21]. The 60-pages illustrated Appendix of the aforementioned companion paper consists of the protocols for all the regions.

In the remainder, the brain atlases are designated as ALBERTs; ALBERTs having been registered onto other individual brain MRIs via their underlying MRI and MRI-to-MRI registrations as “transformed ALBERTs”; average greyscale MRIs as “Templates”; fused atlases in a template space (i.e. in an average space as opposed to an individual space) as maximum probability neonatal ALBERT (MPNA).

### Automatic Segmentation via Multiple ALBERTs

#### Registration

After pre-processing, every neonatal subject was paired with every other neonatal subject for image registration, resulting in 380 (20×19) image pairs. All pairs were aligned using 3D voxel-based registration in three steps using IRTK Software (available via http://wwwhomes.doc.ic.ac.uk/~dr/software/): rigid, affine and non-rigid registration. Parameter settings were tuned to the specific challenges posed by images of neonates [17]. Blurring of both target and source images during the subsequent affine registration improved results. Furthermore, we increased the resolution levels from one to three, increased the number of iterations from 100 to 200, decreased the length of steps from 3.2 to 2 and used the correlation coefficient as the similarity measure in this step. For the final non-rigid registration, iterations were increased from 10 to 100 compared with adult-to-adult registration. The non-rigid step was based on the manipulation of a free-form deformation represented by displacements on a grid of control points blended using cubic B-splines [29] (available via http://wwwhomes.doc.ic.ac.uk/~dr/software/) and maximizing normalised mutual information (NMI; [30]. The registration was refined in a multi-resolution fashion by stepwise reduction of the control point spacing from 20 mm to 10 mm, 5 mm and finally 2.5 mm. Registration pairs were processed in parallel on a cluster of approximately 400 Linux PCs, controlled by Condor software (Version 6.7.13, http://www.cs.wisc.edu/condor/).

#### Label propagation

The output of the registration of an image pair is a transformation that maps the neonatal source image to the neonatal target image. These transformations were then applied to the ALBERTs using nearest-neighbour interpolation, resulting in 19 individualized propagated transformed ALBERTs for each of the 20 target brains.

#### Decision fusion

Each resulting transformed ALBERT assigns a structure label to every voxel in the corresponding MR image volume. To combine the information from multiple individual label sets into a single segmentation, we applied vote-rule decision fusion. The consensus class of each voxel was defined as the modal value of the distribution of the individual label assignments [31]. This approach yielded good results in our previous studies [14], [16], [17], [26]). In the case of non-unique modes, one of the modal values was assigned at random. Even vs. odd numbers of individual label sets resulted in twice the number of equivocal voxels, but in absolute terms, the fraction was very small (less than 1% of the total number of voxels) [16]. Three versions were created.

First, we created fused atlases for all 20 subjects based on fusion of all remaining 19 transformed ALBERTs (ALBERTs_19). Secondly, for the 15 preterms, we also created fused atlases based on only the remaining 14 preterm transformed ALBERTs (ALBERTs_14_Pre); and finally for the term population (n = 5), we also created fused atlases based on only the remaining four term transformed ALBERTs (ALBERTs_4_Term).

### Automatic Segmentation via Probabilistic Templates and MPNAs

#### Neonatal template creation

**Figure 1:**
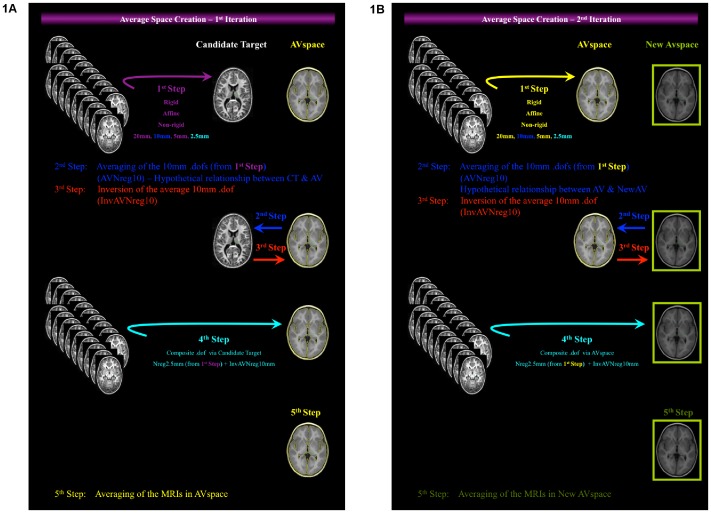
Illustration of the algorithm for the creation of an average space/template, emphasizing schematically on the different spaces involved in the transformations. **1a.** First Iteration: 1st Step: Non-rigid registration of cohort to the candidate target; 2nd Step: Averaging of the nonrigid 10 mm transformation (blue arrow); 3rd Step: Inversion of the average nonrigid 10 mm transformation (red arrow); 4th Step: Composite transformation (nonrigid 2.5 mm+inverted average 10 mm); 5th Step: Averaging of the MRIs in average (AV) space. **1b.** Second Iteration: 1st Step: Non-rigid registration of cohort to the new candidate (AV space); 2nd Step: Averaging of the nonrigid 10 mm transformation (blue arrow); 3rd Step: Inversion of the average nonrigid 10 mm transformation (red arrow); 4th Step: Composite transformation (nonrigid 2.5 mm+inverted average 10 mm); 5th Step: Averaging of the MRIs in New AV space.

One of the term-born controls was selected as the candidate target. All remaining 19 data sets were registered to the candidate target with rigid, affine, and non-rigid registration starting with 20 mm spacing down to 2.5 mm as described above. The process is illustrated in Figure 1. The 10 mm non-rigid transformations were averaged and the average transformation was inverted, on the assumption that this average transformation maps the hypothetical average space we want to create to the candidate target space. Combining each 2.5 mm non-rigid transformation with the inverse average 10 mm non-rigid transformation we transferred each image, through the candidate target space, to the average space. In the second iteration (Figure 1b) we used the mean intensity image in average space as the new candidate target, in order to reduce possible bias arising from the choice of the first candidate target. We registered the 19 data sets to the new candidate average and repeated the steps twice. After the second iteration, we obtained the new average space. Similar approach has been used for the creation of pediatric templates [32].

In order to assess the influence of the bias resulting from the choice of the candidate target on subsequent MPNA registrations we created four different average spaces. For three of these, the visually most representative and symmetrical of the term controls was selected as a candidate target and used to transform 1) the whole cohort, 2) the preterm data sets only, 3) the term data sets only. Finally, we selected the visually most representative and symmetrical preterm data set and transformed only the preterm data sets to this candidate target (Table 1).

**Table 1:** Neonatal Templates based on different candidate target and different prior MRIs.

Template	Candidate Target	Fused information
Template_01	Term-born	All 19 remaining MRIs.
Template_02	Term-born	All 15 preterm MRIs.
Template_03	Preterm	All 14 remaining preterm MRIs.
Template_04	Term-born	All 4 remaining term-born MRIs.

After the creation of the average spaces for the cohort of neonates, each data set was registered to each average space using the same parameter settings as previously and the segmentations were fused to create eight different MPNAs (Table 2), each corresponding to one of the four template spaces (Table 1). This is similar to the creation of a maximum probability atlas for the pediatric population [32].

**Table 2:** MPNAs based on different templates and different ALBERTs.

MPNA	Template	Fused information in a leave-one-out fashion
MPNA_01	Template 01	19 remaining ALBERTs
MPNA_02	Template 02	19 remaining ALBERTs
MPNA_02_Preterms	Template 02	14 remaining preterm ALBERTs
MPNA_03	Template 03	19 remaining ALBERTs
MPNA_03_Preterms	Template 03	14 remaining preterm ALBERTs
MPNA_04	Template 04	19 remaining ALBERTs
MPNA_04_Preterms	Template 04	14 remaining preterm ALBERTs
MPNA_04_Terms	Template 04	4 remaining term ALBERTs

### Validation

Validation of all automatic segmentations was achieved via overlap measurements, expressed as a Dice index (twice the intersection divided by the union; [33], [34]) between the automatically created segmentations and the corresponding manually created ALBERT, which served as the gold standard. Automatic segmentations were based on individual pairwise registrations and subsequent label fusion of:

- all 19 remaining manually created ALBERTs - ALBERTs_19,
- 14 remaining preterms - ALBERTs_14_Pre,
- four remaining terms - ALBERTs_4_Term.

as well as back-registration of the eight MPNAs in the four average template spaces. We also tested the performance of twin pair ALBERT after single label propagation on the corresponding twin brain, compared to the fusion of non-corresponding priors.

We compared the performance of the best methods for each group using two-tailed paired TTEST, after Bonferroni correction for multiple comparisons, with regards to each ROI. For the preterm population we compared the results of ALBERTs_19 with ALBERTs_14 and MPNA_04. For the term-borns we compared the results of ALBERTs_19 with ALBERTs_4 and MPNA_04_Terms. Besides, we used two-tailed paired TTEST to compare the overall performance of these methods for each group.

## Results

**Figure 2:**
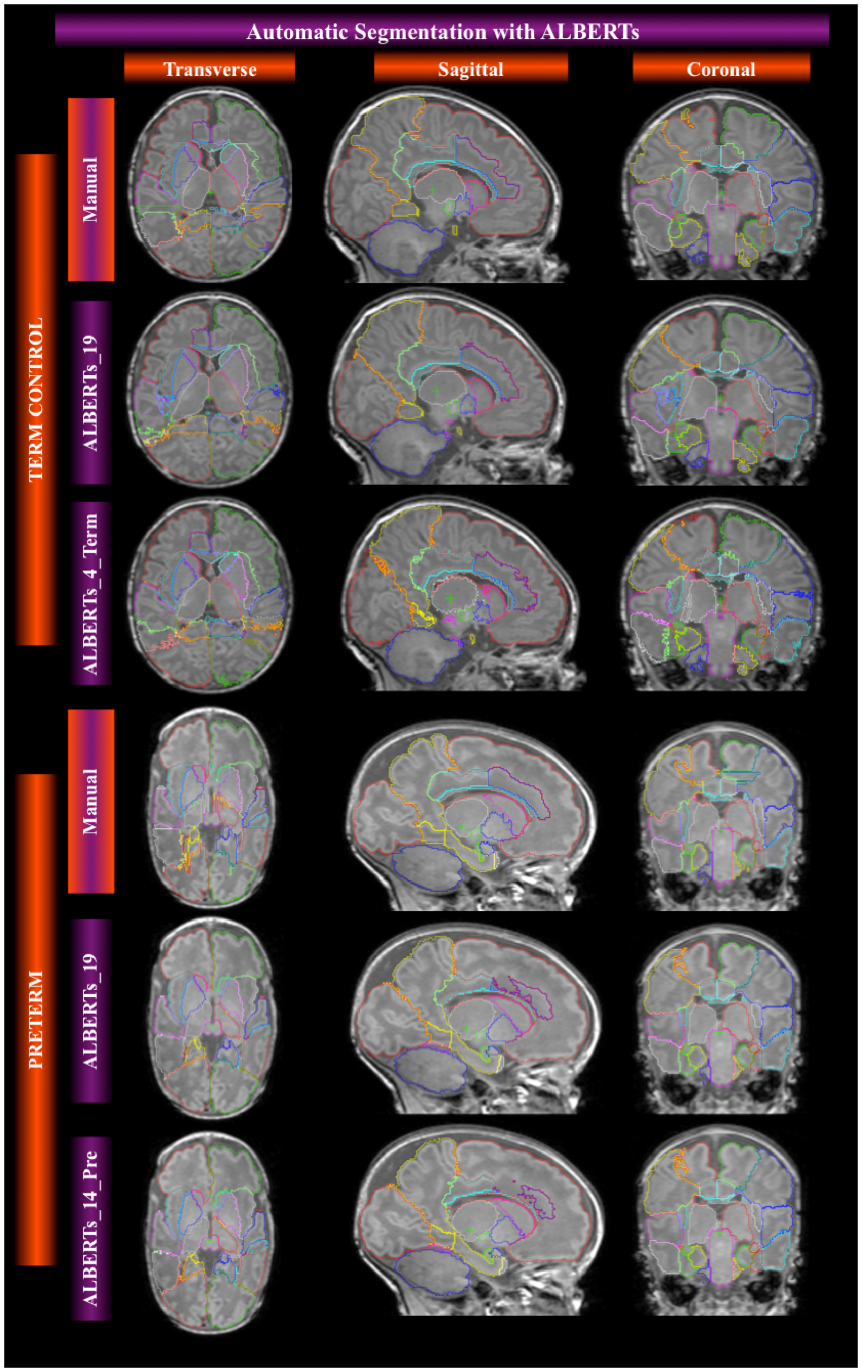
Automatic segmentation using different groups of ALBERTs and decision fusion. Comparison with manual gold standard ALBERT.

A total of forty atlases for 3T MR data sets of neonates resulting from individual pairwise registration and label fusion were created automatically (Figure 2), consisting of 50 ROIs each. Each atlas is the result of label fusion of the remaining 19 ALBERTs (20 atlases), 14 ALBERTs in the cases of preterms (15 atlases), or 4 ALBERTs (5 atlases) in the cases of terms.

**Figure 3:**
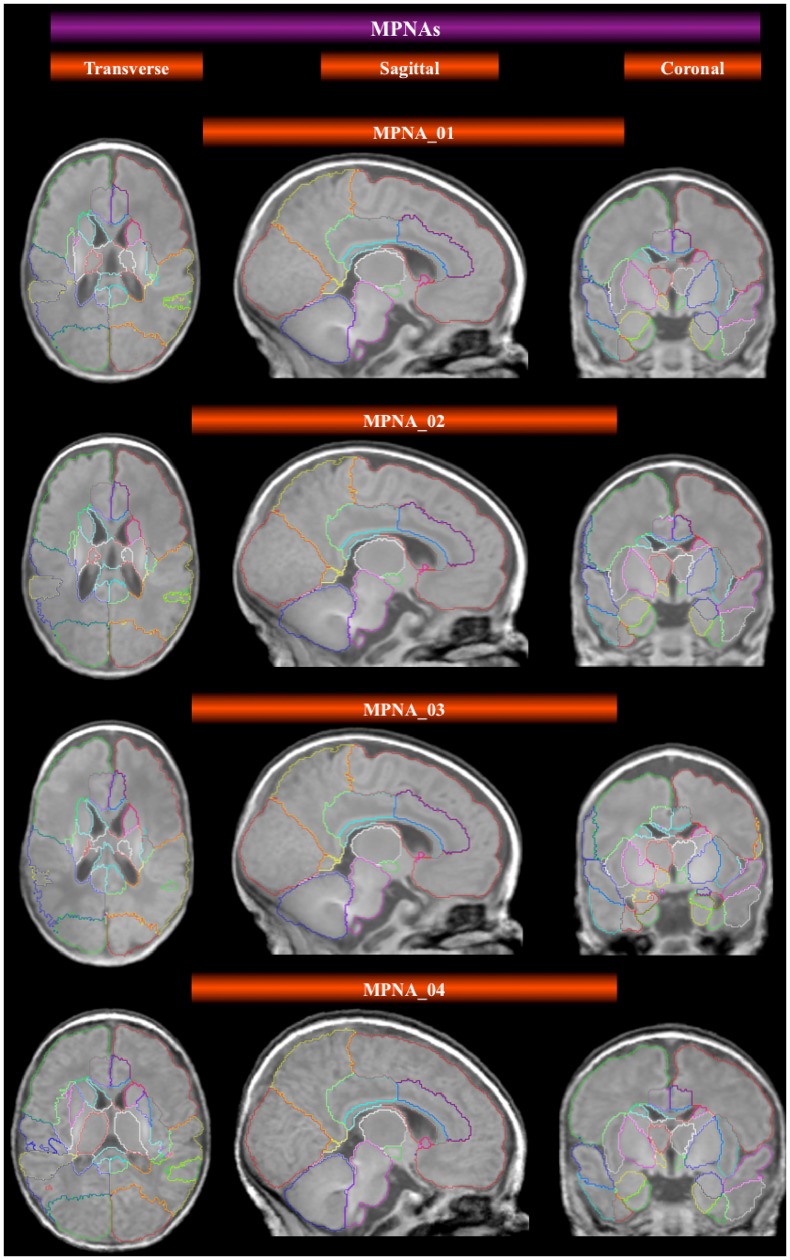
Average space templates and corresponding MPNAs. All MPNAs shown here are derived from fusion of all remaining 19 transformed ALBERTs. Only the template creation differs in terms of the initial candidate target (see Figure 1): term-born for MPNA_01 MPNA_02 and MPNA_04, preterm for 03; and in terms of the MRIs averaged to create the template space: all remaining 19 for MPNA_01; all 15 preterms for MPNA_02; all remaining 14 preterms for MPNA_03, and all remaining 4 terms for MPNA_04.

In the case of template-based segmentations resulting from single registrations of a template to a target, we used the four templates created using different candidate targets and fusing different cohorts (Figure 3) described in Table 1. The eight MPNAs created for the corresponding templates (Figure 3) have been described in Table 2. In total, this resulted in 160 (8×20) individualized segmentations via templates and MPNAs that were compared with their respective manual gold standard.

**Figure 4:**
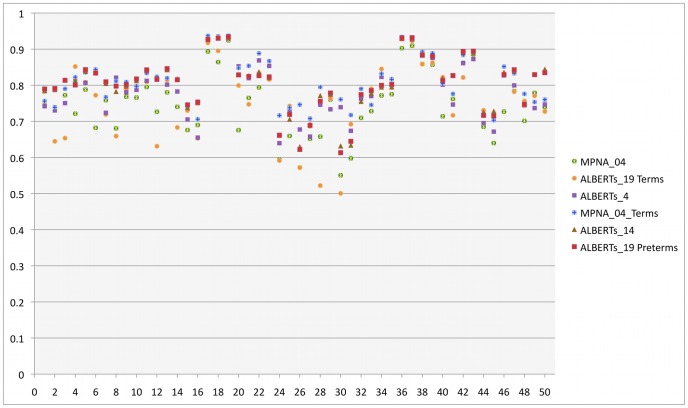
Validation of different approaches for automatic segmentation of the newborn brain. Dice measurements for 50 ROIs, either fusing anatomical prior information from various combinations of ALBERTs or propagating labels of various MPNAs. Only the analytical results of the approaches that performed best are displayed. For translating the numbers into anatomical region names, see Table 4.

Validation was performed by means of Dice measurements. In Table 3 and Figure 4 we display the results of the validation of the different approaches for automatic segmentation when compared with manual gold standards. In Figure 4, we display some comparative Dice measurements for the approaches that performed best, either fusing ALBERTs or using MPNAs. The best methods for each group and the Dice indices for all 50 ROIs are displayed in Tables 4, 5, 6, 7, 8, 9.

**Table 3:** Validation results by means of SI measurements of the different templates, different ALBERTs of Optimum Segmentation and different fusion approaches.

	Term-borns			Preterms		
	Average SI	Stdev	CV (%)	Average SI	Stdev	CV (%)
**MPNAs**						
MPNA_01	0.74	0.02	3	0.73	0.03	4
MPNA_02	0.73	0.02	3	0.72	0.02	3
MPNA_02_Preterms	0.70	0.02	3	0.72	0.02	3
MPNA_03	0.69	0.04	5	0.72	0.03	4
MPNA_03_Preterms	0.66	0.04	5	0.71	0.03	4
MPNA_04	0.75	0.02	3	**0.75** ††	0.03	4
MPNA_04_Preterms	0.72	0.02	3	0.74	0.03	4
MPNA_04_Terms	**0.81** †	0.02	2	0.70	0.03	4
**Fusion of individual transformed ALBERTs**						
ALBERTs_19	**0.76** *	0.03	4	**0.81** **	0.02	3
ALBERTs_4	**0.79** *	0.02	3			
ALBERTs_14				**0.81** **	0.03	4
**Twin ALBERTs**						
ALBERTs_14				0.83	0.01	1
Twin ALBERT Single				0.80	0.002	0.32

SI: Similarity Index, same as Dice Index.

† Significant difference of MPNA_04_Terms with ALBERTs_19 (two-tailed paired TTEST, p<0.001) and ALBERTs_4 (two-tailed paired TTEST, p<0.05).

* Not significant difference with each other.

†† Significant difference of MPNA_04 with ALBERTs_19 and ALBERTs_14 (two-tailed paired TTEST, p<1.0E−07).

** Not significant difference with each other.

**Table 4:** Dice statistics for 50 ROIs with fusion approach ALBERTs_19 for preterms.

No	Name of Structure	Right Hemisphere					Left Hemisphere				
		Mean Dice	SD	CV (%)	Min	Max	Mean Dice	SD	CV (%)	Min	Max
**Temporal Lobe**											
1; 2	Hippocampus	0.79	0.08	10	0.53	0.85	0.79	0.06	7	0.65	0.86
3; 4	Amygdala	0.81	0.04	5	0.75	0.88	0.80	0.04	5	0.72	0.86
5; 6	Anterior temporal lobe, medial part	0.84	0.04	4	0.76	0.90	0.83	0.05	6	0.70	0.88
7; 8	Anterior temporal lobe, lateral part	0.81	0.05	6	0.73	0.89	0.80	0.04	6	0.71	0.85
9; 10	Parahippocampal and ambient gyri ant.p.	0.80	0.06	7	0.67	0.86	0.82	0.03	4	0.74	0.87
25; 24	Parahippocampal and ambient gyri post.p.	0.72	0.05	8	0.60	0.80	0.66	0.08	13	0.43	0.78
11; 12	Superior temporal gyrus, middle part	0.84	0.04	5	0.75	0.89	0.82	0.04	5	0.75	0.88
31; 30	Superior temporal gyrus, post.p.	0.64	0.11	17	0.35	0.78	0.61	0.12	20	0.35	0.78
13; 14	Middle and inferior temporal gyrus ant.p	0.85	0.03	4	0.80	0.88	0.82	0.04	5	0.71	0.87
29; 28	Middle and inferior temporal gyrus post.p.	0.78	0.05	7	0.68	0.85	0.76	0.07	9	0.64	0.84
15; 16	Fusiform gyrus ant.p.	0.75	0.10	13	0.45	0.84	0.75	0.09	13	0.54	0.86
27; 26	Fusiform gyrus post.p.	0.69	0.09	12	0.52	0.80	0.62	0.14	23	0.36	0.78
**Posterior Fossa**											
17; 18	Cerebellum	0.93	0.02	2	0.88	0.95	0.93	0.02	2	0.89	0.96
19	Brainstem	0.93	0.01	1	0.90	0.95	*(unpaired)*				
**Insula and Cingulate gyri**											
21; 20	Insula	0.82	0.05	6	0.71	0.87	0.83	0.05	6	0.72	0.88
33; 32	Cingulate gyrus, anterior part	0.79	0.10	13	0.51	0.88	0.77	0.08	10	0.59	0.88
35; 34	Cingulate gyrus, posterior part	0.80	0.05	6	0.68	0.86	0.80	0.05	6	0.67	0.85
**Frontal Lobe**											
37; 36	Frontal lobe	0.93	0.02	2	0.87	0.95	0.93	0.02	2	0.89	0.95
**Occipital Lobe**											
23; 22	Occipital lobe	0.82	0.07	8	0.68	0.89	0.83	0.05	6	0.69	0.90
**Parietal Lobe**											
39; 38	Parietal lobe	0.88	0.02	2	0.85	0.90	0.88	0.02	2	0.84	0.90
**Basal Ganglia and Thalamus**											
41; 40	Caudate nucleus	0.83	0.04	4	0.73	0.87	0.81	0.05	6	0.71	0.86
43; 42	Thalamus	0.89	0.04	5	0.77	0.93	0.89	0.02	2	0.86	0.92
45; 44	Sub-thalamic nucleus	0.71	0.07	9	0.59	0.82	0.72	0.04	6	0.62	0.77
47; 46	Lentiform nucleus	0.84	0.04	5	0.76	0.91	0.83	0.04	5	0.74	0.88
**Corpus Callosum**											
48	Corpus callosum	0.75	0.04	6	0.65	0.81	*(unpaired)*				
**Ventricles**											
49; 50	Lateral ventricles	0.83	0.03	4	0.77	0.87	0.83	0.04	4	0.77	0.88

**Table 5:** Dice statistics for 50 ROIs with fusion approach ALBERTs_14 for preterms.

No	Name of Structure	Right Hemisphere					Left Hemisphere				
		Mean Dice	SD	CV (%)	Min	Max	Mean Dice	SD	CV (%)	Min	Max
**Temporal Lobe**											
1; 2	Hippocampus	0.78	0.07	9	0.53	0.85	0.75	0.09	12	0.49	0.86
3; 4	Amygdala	0.77	0.09	12	0.54	0.88	0.81	0.04	5	0.72	0.87
5; 6	Anterior temporal lobe, medial part	0.83	0.04	5	0.75	0.90	0.82	0.07	8	0.66	0.89
7; 8	Anterior temporal lobe, lateral part	0.79	0.07	9	0.64	0.89	0.76	0.09	12	0.47	0.85
9; 10	Parahippocampal and ambient gyri ant.p.	0.80	0.05	6	0.67	0.86	0.81	0.05	6	0.66	0.87
25; 24	Parahippocampal and ambient gyri post.p.	**0.72** *	0.05	7	0.60	0.80	0.64	0.10	15	0.42	0.78
11; 12	Superior temporal gyrus, middle part	0.83	0.04	5	0.75	0.89	0.77	0.10	13	0.51	0.88
31; 30	Superior temporal gyrus, post.p.	0.66	0.11	16	0.35	0.78	0.56	0.16	29	0.22	0.78
13; 14	Middle and inferior temporal gyrus ant.p	0.84	0.03	4	0.77	0.88	0.78	0.07	9	0.60	0.87
29; 28	Middle and inferior temporal gyrus post.p.	0.77	0.06	7	0.66	0.85	0.70	0.13	19	0.38	0.84
15; 16	Fusiform gyrus ant.p.	0.74	0.09	12	0.45	0.84	0.73	0.11	15	0.46	0.86
27; 26	Fusiform gyrus post.p.	0.69	0.09	13	0.52	0.80	0.61	0.14	23	0.36	0.78
**Posterior Fossa**											
17; 18	Cerebellum	0.92	0.02	2	0.88	0.95	0.92	0.03	3	0.85	0.96
19	Brainstem	**0.93** *	0.01	1	0.90	0.95	*(unpaired)*				
**Insula and Cingulate gyri**											
21; 20	Insula	0.82	0.05	6	0.72	0.88	0.82	0.05	6	0.72	0.88
33; 32	Cingulate gyrus, anterior part	0.79	0.09	12	0.51	0.88	**0.77** *	0.08	10	0.59	0.88
35; 34	Cingulate gyrus, posterior part	0.81	0.05	6	0.68	0.88	0.81	0.05	6	0.67	0.87
**Frontal Lobe**											
37; 36	Frontal lobe	0.93	0.02	2	0.87	0.95	**0.93** *	0.01	1	0.89	0.95
**Occipital Lobe**											
23; 22	Occipital lobe	0.82	0.06	7	0.68	0.89	0.83	0.05	6	0.69	0.90
**Parietal Lobe**											
39; 38	Parietal lobe	0.88	0.02	2	0.85	0.90	0.88	0.02	2	0.84	0.90
**Basal Ganglia and Thalamus**											
41; 40	Caudate nucleus	0.80	0.07	9	0.61	0.87	0.82	0.04	5	0.71	0.86
43; 42	Thalamus	0.89	0.04	4	0.77	0.93	0.88	0.04	5	0.75	0.92
45; 44	Sub-thalamic nucleus	0.72	0.06	8	0.59	0.82	0.72	0.04	6	0.62	0.77
47; 46	Lentiform nucleus	0.83	0.05	6	0.68	0.91	0.83	0.04	5	0.74	0.88
**Corpus Callosum**											
48	Corpus callosum	0.75	0.04	6	0.65	0.82	*(unpaired)*				
**Ventricles**											
49; 50	Lateral ventricles	0.81	0.06	7	0.64	0.87	0.81	0.07	9	0.59	0.88

* Significant difference (two-tailed paired TTEST, p<0.05) with corresponding value in Table 4. Not significant after correction for multiple comparisons with Bonferroni correction.

**Table 6:** Dice statistics for 50 ROIs with MPNA_04 approach for preterms.

No	Name of Structure	Right Hemisphere					Left Hemisphere				
		Mean Dice	SD	CV (%)	Min	Max	Mean Dice	SD	CV (%)	Min	Max
**Temporal Lobe**											
1; 2	Hippocampus	**0.74** †	0.08	11	0.51	0.82	0.74*	0.05	7	0.64	0.80
3; 4	Amygdala	0.77*	0.03	4	0.70	0.83	0.72*	0.10	13	0.51	0.81
5; 6	Anterior temporal lobe, medial part	**0.79** †	0.06	7	0.64	0.86	**0.68** †	0.12	18	0.49	0.85
7; 8	Anterior temporal lobe, lateral part	0.76	0.07	9	0.64	0.84	0.68*	0.17	36	0.16	0.67
9; 10	Parahippocampal and ambient gyri ant.p.	0.77*	0.06	7	0.61	0.82	**0.77** †	0.03	4	0.71	0.82
25; 24	Parahippocampal and ambient gyri post.p.	**0.66** †	0.07	10	0.54	0.76	0.59*	0.08	14	0.42	0.74
11; 12	Superior temporal gyrus, middle part	0.80*	0.05	6	0.66	0.85	0.73*	0.05	7	0.60	0.79
31; 30	Superior temporal gyrus, post.p.	0.60	0.12	21	0.40	0.78	0.55	0.14	25	0.20	0.69
13; 14	Middle and inferior temporal gyrus ant.p	**0.78** ††	0.03	4	0.73	0.85	**0.74** ††	0.06	8	0.60	0.82
29; 28	Middle and inferior temporal gyrus post.p.	0.76	0.05	7	0.65	0.85	0.66*	0.10	15	0.45	0.75
15; 16	Fusiform gyrus ant.p.	0.68*	0.08	12	0.45	0.78	0.69*	0.08	11	0.55	0.78
27; 26	Fusiform gyrus post.p.	0.65*	0.08	13	0.52	0.79	0.57	0.09	16	0.37	0.70
**Posterior Fossa**											
17; 18	Cerebellum	**0.89** ††	0.03	3	0.84	0.93	**0.86** ††	0.03	3	0.78	0.90
19	Brainstem	**0.92** ††	0.01	1	0.90	0.94	*(unpaired)*				
**Insula and Cingulate gyri**											
21; 20	Insula	**0.76** †	0.06	7	0.60	0.83	0.68*	0.15	22	0.39	0.82
33; 32	Cingulate gyrus, anterior part	0.73	0.09	12	0.55	0.84	0.71*	0.08	12	0.49	0.81
35; 34	Cingulate gyrus, posterior part	0.78	0.05	7	0.68	0.83	0.77	0.04	6	0.70	0.84
**Frontal Lobe**											
37; 36	Frontal lobe	**0.91** ††	0.02	2	0.87	0.93	0.90*	0.03	4	0.81	0.93
**Occipital Lobe**											
23; 22	Occipital lobe	0.82	0.04	5	0.73	0.89	0.79*	0.05	6	0.70	0.87
**Parietal Lobe**											
39; 38	Parietal lobe	0.86*	0.01	1	0.83	0.88	**0.86** ††	0.02	2	0.82	0.88
**Basal Ganglia and Thalamus**											
41; 40	Caudate nucleus	**0.76** ††	0.04	5	0.69	0.82	0.71*	0.10	14	0.51	0.81
43; 42	Thalamus	0.89	0.03	4	0.81	0.92	**0.86** †	0.02	3	0.80	0.90
45; 44	Sub-thalamic nucleus	0.64*	0.09	15	0.41	0.77	0.69	0.07	10	0.54	0.79
47; 46	Lentiform nucleus	**0.78** †	0.06	8	0.63	0.84	0.73*	0.11	15	0.53	0.86
**Corpus Callosum**											
48	Corpus callosum	**0.70** ††	0.05	7	0.58	0.77	*(unpaired)*				
**Ventricles**											
49; 50	Lateral ventricles	**0.78** ††	0.04	5	0.70	0.84	**0.74** ††	0.03	5	0.67	0.80

†† Significant difference (two-tailed paired TTEST, p<0.0001) with corresponding value in Table 4, after Bonferroni correction for multiple comparisons.

† Significant difference (two-tailed paired TTEST, p<0.001) with corresponding value in Table 4, after Bonferroni correction for multiple comparisons.

* Significant difference (two-tailed paired TTEST, p<0.05) with corresponding value in Table 4, after Bonferroni correction for multiple comparisons.

**Table 7:** Dice statistics for 50 ROIs with fusion approach ALBERTs_19 for terms.

No	Name of Structure	Right Hemisphere					Left Hemisphere				
		Mean Dice	SD	CV (%)	Min	Max	Mean Dice	SD	CV (%)	Min	Max
**Temporal Lobe**											
1; 2	Hippocampus	0.75	0.03	4	0.71	0.77	0.64	0.09	14	0.49	0.74
3; 4	Amygdala	0.65	0.09	14	0.54	0.77	0.85	0.01	2	0.84	0.87
5; 6	Anterior temporal lobe, medial part	0.81	0.05	6	0.75	0.85	0.77	0.10	13	0.66	0.89
7; 8	Anterior temporal lobe, lateral part	0.72	0.08	11	0.64	0.82	0.66	0.12	18	0.47	0.79
9; 10	Parahippocampal and ambient gyri ant.p.	0.79	0.02	3	0.76	0.82	0.80	0.08	10	0.66	0.85
25; 24	Parahippocampal and ambient gyri post.p.	0.74	0.02	3	0.72	0.77	0.59	0.12	21	0.42	0.71
11; 12	Superior temporal gyrus, middle part	0.81	0.05	6	0.75	0.86	0.63	0.08	13	0.51	0.72
31; 30	Superior temporal gyrus, post.p.	0.69	0.09	13	0.54	0.77	0.50	0.18	44	0.32	0.73
13; 14	Middle and inferior temporal gyrus ant.p	0.81	0.04	5	0.77	0.86	0.68	0.06	8	0.60	0.74
29; 28	Middle and inferior temporal gyrus post.p.	0.76	0.07	9	0.66	0.83	0.52	0.10	20	0.38	0.64
15; 16	Fusiform gyrus ant.p.	0.73	0.07	10	0.60	0.79	0.65	0.14	22	0.46	0.81
27; 26	Fusiform gyrus post.p.	0.69	0.10	14	0.54	0.78	0.57	0.14	25	0.39	0.72
**Posterior Fossa**											
17; 18	Cerebellum	0.92	0.01	2	0.90	0.94	0.90	0.03	3	0.85	0.93
19	Brainstem	0.93	0.01	1	0.92	0.95	*(unpaired)*				
**Insula and Cingulate gyri**											
21; 20	Insula	0.75	0.06	7	0.68	0.82	0.80	0.04	5	0.74	0.84
33; 32	Cingulate gyrus, anterior part	0.79	0.05	7	0.70	0.83	0.77	0.08	11	0.67	0.85
35; 34	Cingulate gyrus, posterior part	0.82	0.06	7	0.73	0.88	0.85	0.02	3	0.81	0.87
**Frontal Lobe**											
37; 36	Frontal lobe	0.92	0.01	1	0.91	0.93	0.93	0.01	1	0.92	0.94
**Occipital Lobe**											
23; 22	Occipital lobe	0.82	0.03	4	0.79	0.85	0.83	0.04	5	0.77	0.90
**Parietal Lobe**											
39; 38	Parietal lobe	0.86	0.01	2	0.85	0.88	0.86	0.01	1	0.85	0.87
**Basal Ganglia and Thalamus**											
41; 40	Caudate nucleus	0.72	0.08	11	0.61	0.83	0.82	0.03	4	0.78	0.86
43; 42	Thalamus	0.89	0.02	3	0.85	0.91	0.82	0.05	6	0.75	0.86
45; 44	Sub-thalamic nucleus	0.72	0.05	6	0.65	0.75	0.73	0.04	5	0.67	0.77
47; 46	Lentiform nucleus	0.78	0.06	8	0.68	0.84	0.83	0.04	5	0.77	0.88
**Corpus Callosum**											
48	Corpus callosum	0.76	0.05	7	0.67	0.82	*(unpaired)*				
**Ventricles**											
49; 50	Lateral ventricles	0.77	0.09	12	0.64	0.87	0.73	0.10	14	0.59	0.87

**Table 8:** Dice statistics for 50 ROIs with fusion approach ALBERTs_4 for terms.

No	Name of Structure	Right Hemisphere					Left Hemisphere				
		Mean Dice	SD	CV (%)	Min	Max	Mean Dice	SD	CV (%)	Min	Max
**Temporal Lobe**											
1; 2	Hippocampus	0.73	0.07	10	0.51	0.82	0.71	0.08	11	0.55	0.80
3; 4	Amygdala	**0.74** *	0.07	10	0.57	0.83	0.75	0.09	13	0.51	0.85
5; 6	Anterior temporal lobe, medial part	0.79	0.05	7	0.64	0.86	0.72	0.12	17	0.49	0.85
7; 8	Anterior temporal lobe, lateral part	0.74	0.08	11	0.60	0.86	**0.52** *	0.17	33	0.16	0.76
9; 10	Parahippocampal and ambient gyri ant.p.	**0.77** *	0.05	6	0.61	0.82	0.77	0.04	5	0.69	0.82
25; 24	Parahippocampal and ambient gyri post.p.	0.66	0.06	9	0.54	0.76	0.59	0.08	13	0.42	0.74
11; 12	Superior temporal gyrus, middle part	0.80	0.04	6	0.66	0.85	**0.70** *	0.08	11	0.51	0.79
31; 30	Superior temporal gyrus, post.p.	0.62	0.11	19	0.40	0.78	**0.51** *	0.14	28	0.20	0.69
13; 14	Middle and inferior temporal gyrus ant.p	0.79	0.03	4	0.73	0.85	**0.73** *	0.06	8	0.60	0.82
29; 28	Middle and inferior temporal gyrus post.p.	0.76	0.05	7	0.65	0.85	**0.63** *	0.10	16	0.45	0.75
15; 16	Fusiform gyrus ant.p.	0.68	0.08	11	0.45	0.78	0.68	0.08	12	0.54	0.78
27; 26	Fusiform gyrus post.p.	0.66	0.08	13	0.52	0.79	0.58	0.08	15	0.37	0.70
**Posterior Fossa**											
17; 18	Cerebellum	**0.90** *	0.02	3	0.84	0.93	**0.87** *	0.03	4	0.78	0.91
19	Brainstem	0.93	0.01	1	0.90	0.94	*(unpaired)*				
**Insula and Cingulate gyri**											
21; 20	Insula	**0.76** *	0.05	6	0.60	0.83	**0.69** *	0.13	19	0.39	0.82
33; 32	Cingulate gyrus, anterior part	0.73	0.08	11	0.55	0.84	0.73	0.08	11	0.49	0.82
35; 34	Cingulate gyrus, posterior part	0.78	0.05	7	0.68	0.87	0.78	0.04	6	0.70	0.84
**Frontal Lobe**											
37; 36	Frontal lobe	0.91	0.01	2	0.87	0.93	0.91	0.03	4	0.81	0.94
**Occipital Lobe**											
23; 22	Occipital lobe	0.82	0.04	5	0.73	0.89	**0.81** *	0.05	6	0.70	0.89
**Parietal Lobe**											
39; 38	Parietal lobe	0.86	0.02	2	0.83	0.89	**0.86** *	0.02	2	0.82	0.88
**Basal Ganglia and Thalamus**											
41; 40	Caudate nucleus	0.75	0.05	7	0.62	0.82	0.73	0.09	13	0.51	0.84
43; 42	Thalamus	0.89	0.03	3	0.81	0.92	0.85	0.03	4	0.75	0.90
45; 44	Sub-thalamic nucleus	0.65	0.09	13	0.41	0.77	0.69	0.06	9	0.54	0.79
47; 46	Lentiform nucleus	0.78	0.05	7	0.63	0.84	0.75	0.11	14	0.53	0.86
**Corpus Callosum**											
48	Corpus callosum	0.72	0.05	7	0.58	0.80	*(unpaired)*				
**Ventricles**											
49; 50	Lateral ventricles	0.77	0.05	7	0.63	0.84	0.73	0.06	8	0.55	0.82

* Significant difference (two-tailed paired TTEST, p<0.05) with corresponding value in Table 7. Not significant after correction for multiple comparisons with Bonferroni correction.

**Table 9:** Dice statistics for 50 ROIs with MPNA_04_Terms approach for terms.

No	Name of Structure	Right Hemisphere					Left Hemisphere				
		Mean Dice	SD	CV (%)	Min	Max	Mean Dice	SD	CV (%)	Min	Max
**Temporal Lobe**											
1; 2	Hippocampus	0.76	0.03	4	0.70	0.78	0.74	0.08	11	0.60	0.82
3; 4	Amygdala	0.79	0.07	9	0.67	0.84	0.82	0.03	4	0.78	0.86
5; 6	Anterior temporal lobe, medial part	0.84	0.04	5	0.78	0.88	0.84	0.05	5	0.79	0.89
7; 8	Anterior temporal lobe, lateral part	0.77	0.04	5	0.70	0.79	0.81	0.06	8	0.72	0.88
9; 10	Parahippocampal and ambient gyri ant.p.	0.81	0.02	2	0.79	0.84	0.80	0.05	6	0.74	0.85
25; 24	Parahippocampal and ambient gyri post.p.	0.74	0.07	9	0.62	0.79	**0.72** †	0.04	6	0.66	0.76
11; 12	Superior temporal gyrus, middle part	0.83	0.03	3	0.79	0.86	0.82	0.04	4	0.77	0.86
31; 30	Superior temporal gyrus, post.p.	0.72	0.06	8	0.64	0.78	0.76	0.03	4	0.72	0.79
13; 14	Middle and inferior temporal gyrus ant.p	0.82	0.02	3	0.79	0.85	0.82*	0.06	8	0.71	0.87
29; 28	Middle and inferior temporal gyrus post.p.	0.78	0.04	5	0.72	0.82	0.79	0.04	6	0.73	0.85
15; 16	Fusiform gyrus ant.p.	0.74	0.06	8	0.69	0.81	0.71	0.06	9	0.61	0.78
27; 26	Fusiform gyrus post.p.	0.71	0.07	11	0.63	0.80	0.75	0.07	10	0.63	0.82
**Posterior Fossa**											
17; 18	Cerebellum	0.94	0.01	1	0.91	0.95	0.94	0.02	2	0.90	0.95
19	Brainstem	0.94	0.01	1	0.93	0.94	*(unpaired)*				
**Insula and Cingulate gyri**											
21; 20	Insula	0.85	0.01	2	0.84	0.87	0.85	0.02	2	0.83	0.87
33; 32	Cingulate gyrus, anterior part	0.75	0.06	8	0.65	0.81	0.79	0.05	6	0.71	0.82
35; 34	Cingulate gyrus, posterior part	0.82	0.04	6	0.75	0.86	0.83	0.02	3	0.79	0.86
**Frontal Lobe**											
37; 36	Frontal lobe	0.93	0.01	1	0.91	0.94	0.93	0.00	1	0.93	0.94
**Occipital Lobe**											
23; 22	Occipital lobe	0.87	0.04	5	0.79	0.90	0.89	0.01	1	0.88	0.91
**Parietal Lobe**											
39; 38	Parietal lobe	0.89	0.01	1	0.88	0.90	0.89*	0.01	1	0.87	0.90
**Basal Ganglia and Thalamus**											
41; 40	Caudate nucleus	0.78	0.04	5	0.73	0.81	0.80	0.04	5	0.77	0.86
43; 42	Thalamus	0.90	0.02	2	0.87	0.92	**0.88** †	0.03	4	0.84	0.92
45; 44	Sub-thalamic nucleus	0.70	0.05	6	0.65	0.78	0.72	0.05	7	0.63	0.75
47; 46	Lentiform nucleus	**0.83** †	0.05	6	0.77	0.88	0.85	0.03	3	0.81	0.88
**Corpus Callosum**											
48	Corpus callosum	0.78	0.03	3	0.74	0.80	*(unpaired)*				
**Ventricles**											
49; 50	Lateral ventricles	0.75	0.08	11	0.64	0.83	0.76	0.08	10	0.63	0.84

* Significant difference (two-tailed paired TTEST, p<0.05) with corresponding value in Table 7, after Bonferroni correction for multiple comparisons.

† Significant difference (two-tailed paired TTEST, p<0.05) with corresponding value in Table 8, after Bonferroni correction for multiple comparisons.

We compared the performance of the best methods for each group using two-tailed paired TTEST, after Bonferroni correction for multiple comparisons, with regards to each ROI. For the preterm population we compared the results of ALBERTs_19 with ALBERTs_14 and MPNA_04. For the term-borns we compared the results of ALBERTs_19 with ALBERTs_4 and MPNA_04_Terms. Two-tailed paired TTEST showed that the overall performance of ALBERTs_19 and ALBERTs_14 was significantly better than MPNA_04 (Table 3). Also, for the term population, MPNA_04_Terms performed significantly better than ALBERTs_19 and ALBERTs_4 (Table 3).

For the preterms, in a regional level, ALBERTs_19 performed similarly to ALBERTs_14, without significant differences after Bonferroni correction for multiple comparisons (Tables 4–5). ALBERTs_19 performed better than MPNA_04 in all the regions in either one or both hemispheres, apart from the posterior part of the superior temporal gyrus and the posterior part of the cingulate gyrus (Tables 4, 6).

For the term-borns, in a regional level, ALBERTs_19 performed similarly to ALBERTs_4, without significant differences after Bonferroni correction for multiple comparisons (Tables 7–8). MPNA_04_Terms performed better than ALBERTs_19 in the anterior part of the middle and inferior temporal gyrus and the parietal lobe (Tables 7, 9). MPNA_04_Terms performed better than ALBERTs_4 in the posterior part of the parahippocampal gyrus, the thalamus and the lentiform nucleus (Tables 8–9).

**Figure 5:**
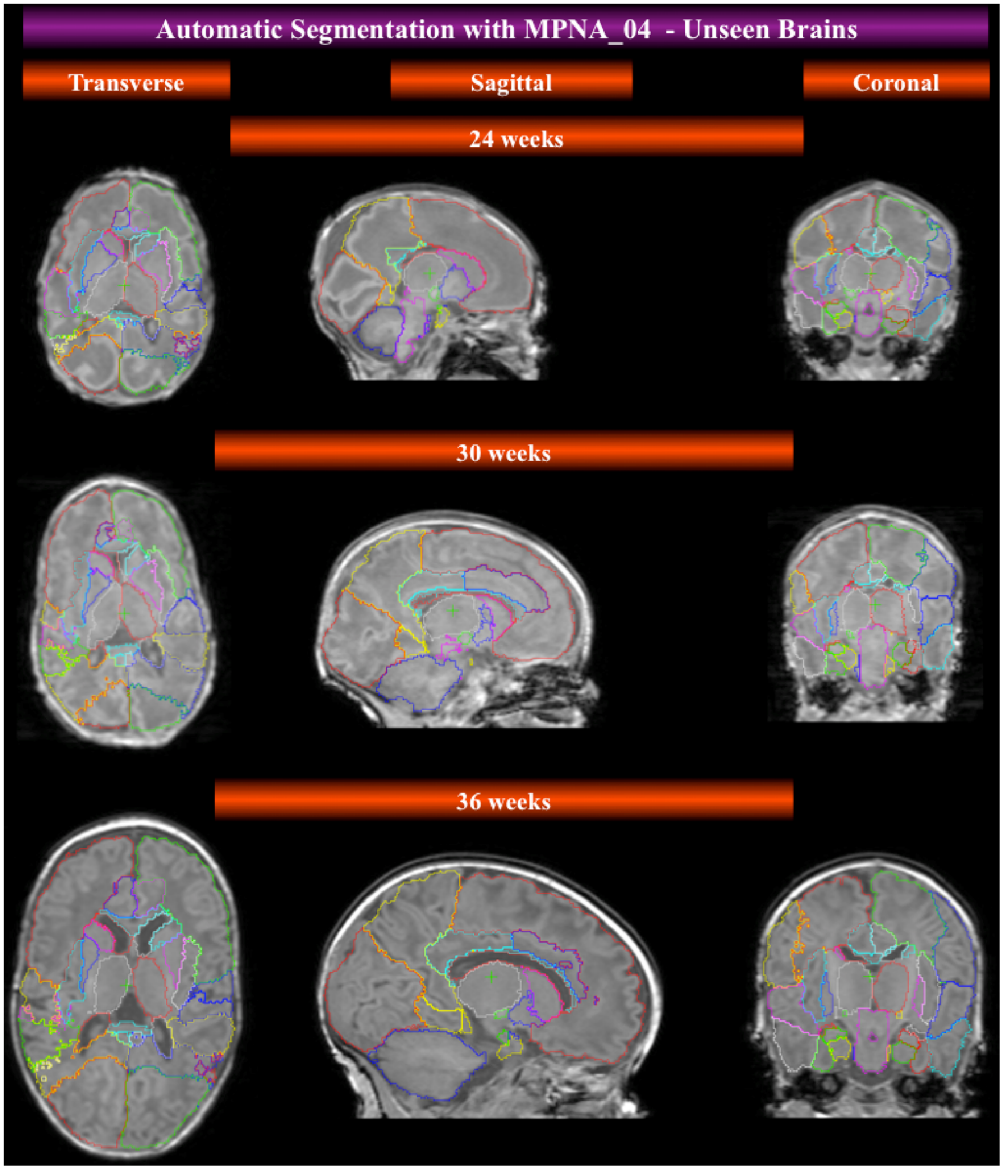
Performance of MPNA_04 in the automatic segmentation of three randomly chosen unlabeled developing brains at various ages, which did not form part of the cohort of priors. The segmentation is the result of a single step registration and propagation of the MPNA.

In Figure 5 we display the preliminary results of an automatic segmentation of developing brain MRIs that do not belong to the cohort of twenty used for the creation of the manually defined ALBERTs. In this instance, automatic segmentation was achieved via a single registration of a template constructed from term-borns (Template_04), whereas the MPNA was obtained through fusion of all ALBERTs transformed into the space of Template_04 (MPNA_04, see Tables 1 and 2). The results are visually acceptable.

ALBERTs and MPNAs with corresponding MRIs and templates will become available through our website www.brain-development.org.

## Discussion

We present two methods for automatic segmentation of neonatal brain MR images into 50 ROIs. The first approach is based on fusion of anatomical prior information from various manually constructed neonatal atlases after one pairwise registration per atlas used. The second approach is based on propagation of labels from various neonatal MPNAs, requiring only one registration. In both cases we evaluated the performance of different selections neonatal atlases and MPNAs obtained via different strategies. The approaches were compared with the manual “gold standard” segmentations by means of the Dice overlap coefficient. The maximum Dice values obtained, averaged across all regions, were 0.81±0.02 using label propagation and fusion for the preterm population, and 0.81±0.02 using the single registration of a MPNA based on term controls only, in combination with a template based on term controls only, too. Such Dice overlaps are in line with results using maximum probability maps in adults, and somewhat lower than multi-atlas propagation and label fusion in adults [14]. Segmentations of unlabeled ex-cohort target images yielded segmentations of high quality on visual inspection.

In terms of atlases used, pre-processing pipeline and parameters used, we present the first detailed evaluation of several strategies for the automatic segmentation of neonatal and developing brains. This was made possible through the availability of manual priors. It took 18 person-months to delineate the 1000 (50×20) structures and thus create the first cohort of neonatal manual priors, and another four to check them for consistency with the protocols; it is unlikely that larger single-investigator datasets will ever become available. The ALBERTs will become available through our website (www.brain-development.org). While validation of automatic labeling methods is only possible within-sample, where labels created manually with the same protocol are available for calculating overlaps, the availability of the atlases will make it possible to assess automatic segmentation of other cohorts, e.g. NIHPD (http://pediatricmri.nih.gov/nihpd).

The validation of the two different methods is based on leave-one-out approaches, which have been widely used by researchers in the past, including our team [14], [16], [26], [35], [36]. In such an approach, for the validation of the ALBERTs_19 performance for example, for each of the target brains we use as priors the remaining 19. This means that the manual segmentation of the target brain and the automatic segmentation we obtain after registration, propagation and fusion are two totally independent segmentations. The results of the validation by all means highlight the potential to segment unseen brains.

Automatic segmentation is commonly used in studies of adult MR brain images but has been challenging in infants. An initial spatial normalization to a template or average brain in a standard stereotaxic space [37], [38] can be problematic. Spatial normalization requires an appropriate template [39], and when cerebral images of children are aligned using an adult template the variation of anatomical landmarks is increased [40], [41], and greater nonlinear local deformations are required for registration [42]. Indeed the use of the adult MNI template [43] in infants and children has been criticized [44], [45], [46], and pediatric templates recommended for the analysis of pediatric images [47], [48], [49]. Transforming neonatal rather than pediatric cerebral images to an adult template has additional difficulties [37] including age-dependent differences in regional brain size [50] and unmyelinated white matter with different MR characteristics in neonates [51], so that several groups have constructed specific neonatal templates [37], [52], [53], [54], [55], [56].

Tissue segmentation is also difficult due to the different and highly variable tissue characteristics [18], [57]. Prastawa et al. (2005) reported an automated method using a three-subject atlas for GM, CSF and myelinated and unmyelinated WM, but did not attempt subcortical GM segmentation [20]. Warfield et al. (2000) use a specific template for newborn brains with predefined classifications for myelinated and unmyelinated WM [58]. Huppi et al. (1998) and Inder et al. (2005) showed tissue class segmentation results of newborn infants using this method [59], [60]. Kazemi et al. (2011) presented a neonatal brain phantom that consists of 9 different tissue types: skin, fat, muscle, skull, dura mater, gray matter, myelinated white matter, nonmyelinated white matter and cerebrospinal fluid [61].

Despite the difficulties there have been previous reports of automatic segmentation methods for newborn infants. Nishida et al. (2006) presented a semi-automated method for segmentation of preterm infants at term corrected age into anatomical ROI. Unfortunately, their cohort did not include any term controls and they did not validate with any gold standard segmentation and hence the comparison with our method is difficult [62]. There are also approaches based on Diffusion Tensor Imaging, resulting in segmentations with numerous regions with or without clear anatomical or functional correspondence [63], [64]. These approaches yield results that are visually plausible, but have not yet been compared or validated against external standards in the neonatal population, as for example defined anatomical protocols. It is hence difficult to compare our work with these two studies.

The average spaces required for spatial normalisation were created using an approach similar to that of Guimond et al. (2000) and Rueckert et al. (2003) for averaging local deformations [39], [65], also used in the pediatric population [32]. In some studies, contrary to the main trend of using a standard reference template like MNI, a single subject data set of the image group is selected as the reference or template image [66], [67], [68]. A disadvantage of this atlas construction method is that the resulting atlas can inherently contain unique features of the selected initial reference image, which results in local topological bias [69]. Group-wise registration, based on the minimization of the average deformation field, could be a solution to the problem [70], [71]. However, the presence of a few very unusually shaped brains (cf. Figure 1 of Gousias et al. (2012) [21]) coupled with the small number of subjects available due to the phenomenal effort required for manual delineation, leads us to believe that our strategy of explicitly choosing “normal looking” brains is appropriate in this situation. Extremely dolichocephalic subjects or subjects with obvious major asymmetries were not selected as candidate targets. Group-wise registration remains an area for future study.

The average Dice indices for the various approaches (Table 3) and the Dice indices for the best approaches (Tables 4, 5, 6, 7, 8, 9) indicate that fusing ALBERTs of the same group (in terms of degree of myelination) yields better or similar results than fusing more classifiers from different groups: term priors performed better for a term target and preterm priors better for a preterm target, confirming our previous findings [17]. Also, single propagations from twin pair, expected to be more similar than brain MRIs from unrelated subjects, perform at a level comparable to fusion. This finding highlights the importance of resemblance in sulcal and gyral patterns between the source and the target brain, as it has been shown before in corresponding scans between different ages in the context of longitudinal segmentation [17]. Optimal template selection approaches have previously been shown to be effective in atlas-based segmentation of confocal microscopy images of bee brains [15], as well as in human brain segmentation [72], [73].

An MPNA was created for the term (MPNA_04_Term) and the preterm brain (MPNA_04_Preterm). This type of atlases [26], [32] has shown its potential in the absence of a bigger database or for computational time savings [14]. In the present study, their application results in segmentation accuracies comparable with the segmentation using fusion of transformed ALBERTs. The results of the MPNA_04 template registration, between source template and target image, show the need for crispier and not extremely smooth templates (MPNA_02, 03), which incorporate the basic anatomical information from a smaller number of images and not necessarily the whole cohort (MPNA_01). Besides, the results illustrated in Figure 5 highlight the effectiveness of the MPNA_04 template registration through its intrinsic smoothness to capture the lack of prominent cortical anatomical landmarks in the extremely preterm population. The latter findings highlight the importance of the feature of smoothness, which has to be present but not to an extreme level. Template selection is important, because it has been shown that the choice of the template affects region-based volumetric analysis, either when the template does not correspond to the age cohort [49] or when multiple templates are used [74].

In neonates, the selection of the candidate target was also based on symmetry and normality criteria. The first template (term candidate target – all subjects) is slightly rounder on transverse sections than the second (term candidate target – preterms) (Figure 3). This happens because the term brains seem to have a more round/spherical brain shape. The difference between the second template and the third (preterm candidate target – preterms) is more obvious, especially in the subcortical tissues, because of the different candidate target (Figure 3). This could indicate that a good template, in terms of representation of anatomy and corresponding tissue properties, should be limited to a cohort of data sets of tight gestational age range, due to the rapid progression of myelination of the WM and the contrast issues arising as a consequence. In case of a wider gestational age range the template may become extremely blurry. This may be the reason template 3, which was based on the whole preterm cohort and not some images of tighter age range, did not perform as expected for the corresponding preterm population. The fact that template 04, based on a term control candidate average and MRI averaging of the transformed ALBERTs of the remaining four term controls (ALBERTs_4_Term), gave the best results for the term population also supports this statement.

Atlases containing such detailed segmentation can be useful in the monitoring of developmental growth of different brain regions in longitudinal studies or aid group comparisons between normal controls and pathological cases. The associated templates can be used as a reference in functional and connectivity studies and will benefit from the anatomical annotations contained in the associated MPNAs. Both methods presented here yield very plausible and comparable results, ALBERTs performing slightly better in absolute Dice measurements for the preterm. However, MPNAs have the advantage of requiring only one registration per target brain and will require fewer computational power resources (8 hours compared to 20×8 = 160 hours for all ALBERTs, even if the latter process can be calculated in parallel on a cluster of computers).
